# Efficacy of high-intensity focused ultrasound-assisted hepatic resection (HIFU-AR) on blood loss reduction in patients with liver metastases requiring hepatectomy: study protocol for a randomized controlled trial

**DOI:** 10.1186/s13063-017-1801-2

**Published:** 2017-02-06

**Authors:** Aurélien Dupré, David Pérol, Ellen Blanc, Patrice Peyrat, Valéria Basso, Yao Chen, Jérémy Vincenot, Anthony Kocot, David Melodelima, Michel Rivoire

**Affiliations:** 1 Department of Surgical Oncology, Centre Léon Bérard, 28 Rue Laennec, Lyon, 69008 France; 2 Inserm, U1032, LabTau, University of Lyon, Lyon, 69003 France; 3 Department of Clinical Research (DRCI), Centre Léon Bérard, Lyon, 69008 France

**Keywords:** High-intensity focused ultrasound, HIFU, Liver surgery, Hepatectomy, Liver metastasis, Liver transection, Blood loss, Blood transfusion, Randomized trial

## Abstract

**Background:**

Liver resection is the only potentially curative treatment for colorectal liver metastases (LM). It is considered a safe procedure, but is often associated with blood loss during liver transection. Blood transfusions are frequently needed, but they are associated with increased morbidity and risk of recurrence. Many surgical devices have been developed to decrease blood loss. However, none of them has proven superior to the standard crushing technique. We developed a new, powerful intra-operative high-intensity focused ultrasound (HIFU) transducer which destroys tissue by coagulative necrosis. We aim to evaluate whether HIFU-assisted liver resection (HIFU-AR) results in reduced blood loss.

**Methods:**

This is a prospective, single-centre, randomized (1:1 ratio), comparative, open-label phase II study. Patients with LM requiring a hepatectomy for ≥ 2 segments will be included. Patients with cirrhosis or sinusoidal obstruction syndrome with portal hypertension will be excluded. The primary endpoint is normalized blood loss in millilitres per square centimetre of liver section plane. Secondary endpoints are: total blood loss, transection time, transection time per square centimetre of liver area, haemostasis time, clip density on the liver section area, rate and duration of the Pringle manœuvre, rate of patients needing a blood transfusion, length of hospital stay, morbidity, patients with positive resection margin, and local recurrence. Assuming a blood loss of 7.6 ± 3.7 mL/cm^2^ among controls, the study will have 85% power to detect a twofold decrease of blood loss in the experimental arm, using a Wilcoxon (Mann-Whitney) rank-sum test with a 0.05 two-sided significance level. Twenty-one randomized patients per arm are required. Considering the risk of contraindications at surgery, up to eight patients may be enrolled in addition to the 42 planned, with an enrolment period of 24 months. Randomization will be stratified by surgeon.

**Discussion:**

We previously demonstrated the safety and efficacy of intra-operative HIFU in patients operated on for LM. We also demonstrated the efficacy of HIFU-AR in a preclinical study. Participants in the HIFU-AR group of this randomized trial can expect to benefit from reduced blood loss and decreased ischemia of liver parenchyma.

**Trial registration:**

Clinicaltrial.gov, NCT02728167. Registered on 22 March 2016.

**Electronic supplementary material:**

The online version of this article (doi:10.1186/s13063-017-1801-2) contains supplementary material, which is available to authorized users.

## Background

### Medical and scientific context

Patients with most solid tumours may develop liver metastases (LM). However, LM occur mainly in patients with colorectal cancer (CRC). In France, CRC is the second most frequent cancer, with an incidence of nearly 41,000 cases each year. It continues to have a poor prognosis and causes nearly 16,000 deaths per year, often due to LM. Hepatic resection is the mainstay of treatment for LM and remains the only potentially curative intervention.

Unfortunately, LM resections are complex major procedures and the resulting blood loss may be significant, leading to the requirement for blood transfusions during the procedure. Such transfusions are correlated with increased morbidity, mortality and risk of tumour recurrence [1–4]. Therefore, the control of blood loss is mandatory to limit morbidity, mortality and recurrence.

Since the hepatic parenchyma tolerates ischemia better than bleeding, intra-operative manœuvres have been proposed to minimize the risk of bleeding during hepatic resection. These include total vascular exclusion, the Pringle manœuvre and selective ligature to control hepatic inflow [5–10]. None of these methods is universally practical or completely effective, and each carries a risk of inducing liver dysfunction in patients with chronic liver disease. Vascular clamping is a time-critical technique, since the risk of morbidity is increased if clamping lasts more than 60 continuous minutes.

### Overview of high-intensity focused ultrasound (HIFU)

HIFU is mechanical waves generated by an ultrasound transducer. As with conventional ultrasound, HIFU can propagate harmlessly through living tissue. However, if the ultrasound beam carries sufficient energy and is brought into a tight focus, the energy within the focal volume can cause a local rise in temperature of sufficient magnitude (up to 80 °C) to generate irreversible cell death. This occurs in a few seconds through coagulative necrosis without damaging the surrounding tissue.

HIFU offers several advantages, which encourage its clinical development. First, HIFU allows non-invasive real-time targeting using sonography. Such real-time monitoring enables adjustment of treatment plans. Second, the mechanism of action of HIFU is not tumour-specific; therefore a wide range of tumour types can be targeted. Third, HIFU is mechanical waves, therefore there is no ionizing radiation. Treatment can be given more than once, as there is no upper limit of tissue tolerance to repeated ultrasound exposure. Finally, HIFU generates very few side effects, and serious adverse events are rare [11]. In several centres worldwide, HIFU has been in clinical use for 5 to 10 years, sometimes in daily practice, to treat solid tumours (both malignant and benign), including those of the prostate [12], liver [13, 14], breast [15] and kidney [16]. Current data are encouraging and the role of HIFU in oncology is likely to expand as devices become more widely available.

However, several technical limitations of currently available HIFU devices have been raised, such as the long time needed to treat tumours of several cubic centimetres. For example, the sonication time (defined as the time from the first to the last sonication) to ablate abdominal pathologies such as uterine leiomyomas with target volumes of 4.6 cm^3^ ranged from 32 to 135 minutes [17, 18]. The use of an extracorporeal HIFU device is clinically feasible for the treatment of hepatocellular carcinoma [13, 19] but only a small part (approximately 30%) of the liver is accessible using a non-invasive HIFU device. In addition, phase aberration and liver movements can produce secondary lesions (such as skin burns or gastric lesions) if a completely extracorporeal treatment is performed. To fill these technical gaps, we undertook a research program on CRC LM treatment by HIFU.

### The HIFU toroidal device: intra-operative HIFU prototype

We previously developed a toroidal HIFU transducer that enables the destruction of large volumes of tissue over short periods of time [20]. This work led to the development of a prototype that has been tested in vitro and in preclinical studies. These studies demonstrated the safety and efficacy of treating the liver using HIFU generated by a toroidal transducer [21–26]. A collaboration with the EDAP TMS company has allowed a prototype to be developed for clinical use and this is currently being evaluated for the treatment of colorectal LM [27].

The HIFU probe is brought into contact with the liver using a sterile ultrasound cooling and coupling liquid (Ablasonic®, EDAP TMP, Vaulx en Velin, France) contained in a sterile polyurethane envelope (Civco, Kalona, IA, USA). It is possible to change the location of the focal zone by adjusting the quantity of coupling liquid between the device and tissues or by electronic focusing. Under these conditions, the focal zone can be placed anywhere between the surface of the liver and 9 cm in depth. This allows treatment of the entire plane of liver transection. A 7.5 MHz ultrasound imaging probe (Vermon, Tours, France) is placed in the centre of the device and connected to a BK HAWK 2102 EXL scanner (BK Medical, Herlev, Denmark) to guide the liver resection. Specific software is used to define the treatment zone from a two-dimensional ultrasound image. The user interface displays the position of the HIFU-treated region superimposed on the sonogram obtained by the integrated ultrasound imaging probe. It is therefore possible to locate the ablation and visualize in real time the treated zone created during ultrasound exposures.

### Previous clinical results

Our team has already demonstrated the safety and feasibility of HIFU for intra-operative ablation of liver parenchyma. We have also demonstrated in an animal study that HIFU can be used to assist hepatic resection.

The HIFU device using a toroidal HIFU transducer guided by ultrasound imaging was evaluated clinically for the treatment of colorectal LM during an open procedure. The transducer has a toroidal shape with a diameter of 70 mm and is divided into 256 emitters each of 0.13 cm^2^. The radius of curvature is 70 mm to enable treatment of the deepest regions of the liver. The operating frequency is 3 MHz.

We conducted a prospective, single-centre phase I–IIa trial [27]. HIFU was delivered immediately before scheduled hepatectomy. To demonstrate the safety and efficacy of rapidly ablating large liver volumes, ablations were performed on healthy tissue within the areas scheduled for resection. In total, 30 ablations (one superficial and one at least 1 cm deep) were carried out in 15 patients. These ablations were all generated within 40 seconds and on average measured 27.5 × 21 mm. The phase I element of the study (n = 6) showed that use of the HIFU device was feasible and safe, and did not damage neighbouring tissue. The phase IIa study (n = 9) showed both that the area of ablation could be precisely targeted on a previously implanted metallic mark and that ablations could be undertaken deliberately to avoid such a mark. Ablations were achieved with a precision of 1–2 mm.

These results enable us to conduct a randomized phase II trial to evaluate whether HIFU-assisted liver resection (HIFU-AR) reduces blood loss compared to standard liver resection in patients with LM.

## Methods/Design

### Objectives

The primary objective is to compare the blood loss during hepatectomy in patients treated by HIFU-AR (arm A) versus standard liver resection (arm B).

The primary endpoint is normalized blood loss in millilitres/square centimetres, defined as total blood loss (in millilitres) divided by the resected hepatic surface (in square centimetres). Total blood loss will be measured during the whole liver transection procedure, i.e. between beginning the section of liver parenchyma and the end of resection including complete haemostasis of the raw surface of the remaining liver. In arm A, time needed to perform HIFU pre-coagulation will be added to this duration. Of note, blood loss is systematically determined by weighing all blood-soaked surgical mops (PB1502, Mettler Toledo, Columbus, OH, USA) and the aspirated fluid (blood suctioned out of the abdomen) on a high-precision scientific scale.

In both arms, the secondary endpoints are:

- transection time (minutes) defined as the time required to achieve the liver transection (minutes) and the transection time per surface of resected liver area (minutes/square centimetres);
- haemostasis time (minutes), defined as the time lapse between the end of transection until completion of haemostasis (minutes);
- clip density on the section area, defined as the total number of haemostatic clips used to perform haemostasis on the liver section area divided by the raw surface of the remaining liver (in square centimetres) (number of clips per square centimetre of liver area);
- rate and duration of the Pringle manœuvre, defined as the number of patients requiring Pringle’s manœuvre during the operating procedure [if blood loss > 500 mL during the transection, measured through the suction device, the surgeon will be authorized to perform a hepatic pedicular clamping (Pringle’s manœuvre)];
- rate of patients needing a blood transfusion, defined as the number of patients requiring blood transfusions either during the operation or after it (per-operative transfusion will be required for patients with blood loss > 1000 mL; post-operative transfusion will be required if Hb <8 g/dL or 10 g/dL if previous history of coronary disease or stroke);
- duration of hospital stay (days), calculated from D_0_ (date of surgery) to the end of hospitalization post-surgery;
- safety and tolerability assessed using the Dindo-Clavien classification specific to surgical complications. During the first 10 days after surgery, we will collect data on haemoglobin, platelets, alanine amino-transferase (ALT), aspartate amino-transferase (AST), gamma-glutamyltransferase (GGT), prothrombin time (PT) and total bilirubin values;
- the proportion of patients with malignant tumours who have a positive resection margin and patients with local recurrence (from randomization to M_6_).

### Study design

This is a prospective, single-centre, randomized, comparative, open-label, phase II study to evaluate the efficacy and the safety of HIFU-AR, using the device described, during an open liver resection in patients with LM.

Patients will be randomized 1:1 to either:

- arm A (experimental arm): pre-coagulation by HIFU-AR plus standard surgical liver resection
- arm B (control arm): standard surgical liver resection.

The randomization will be performed via an Internet platform in the operating room and will be stratified by surgeon (AD, MR, or PP) to ensure equal allocation of subgroups of participants (i.e. to each surgeon) in each arm.

**Fig. 1:**
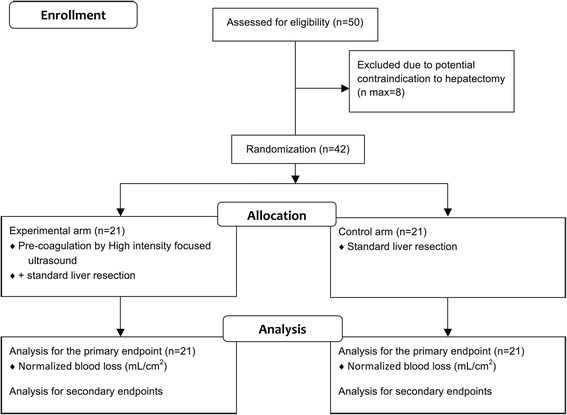
CONSORT flowchart

The flowchart is represented in Fig. 1. The Standard Protocol Items: Recommendations for Interventional Trials (SPIRIT) checklist can be found in Additional file 1.

### Study population

Inclusion and exclusion criteria are listed in Table 1.

**Table 1:** Inclusion and exclusion criteria

Inclusion criteria	Exclusion criteria
Age ≥ 18 years	History of previous major hepatic surgery (i.e. more than three liver segments) or biliary major surgery (except gall-bladder surgery by laparoscopy if more than 6 months previously)
Patients with liver metastases requiring a hepatectomy of ≥ 2 segments	Patients with cirrhosis or sinusoidal obstruction syndrome with portal hypertension
Eastern Cooperative Oncology Group performance status ≤ 1	Pregnant women
Adequate bone marrow and liver function at baseline (defined by platelet count ≥ 100 × 10^9^/L, and haemoglobin of ≥ 9 g/dL, and total bilirubin ≤ 1.5 × upper limit of normal, and transaminases ≤ 5× upper limit of normal)	
Recovered from prior anti-neoplasic treatment-related toxicity (grade <2 persistent treatment-related toxicity as per Common Terminology Criteria for Adverse Events v4 are accepted	
Willingness for follow-up visits	
Covered by a medical insurance	
Signed and dated informed consent indicating that the patient has been informed of all aspects of the trial prior to enrolment	

### Study medical device

The study device is the HIFU prototype from the EDAP TMS company. Currently, this device has no CE mark and is restricted to clinical trials.

The HIFU system is composed of three parts:

- a sterilisable probe, manually brought into contact with the liver, and its accessories (sterile envelope and O-ring seal);
- a treatment module including electrical signal generation and treatment control systems. HIFU exposures are activated using a foot pedal.
- LivKit: single-use consumable

A sterile envelope (Civco #610-542) is placed around the probe to create a reservoir that will be filled by a liquid (Ablasonic®; EDAP TMS, Vaulx-en-Velin, France) ensuring ultrasonic coupling and the transductor’s cooling. The O-ring seal keeps the balloon in place and ensures the reservoir is sealed. The single-use LivKit is installed on the HIFU system before each treatment. During treatments, the transducer is cooled using a continuous flow of Ablasonic® at 10 °C at a rate of 0.3 L/min. A peristaltic Masterflex pump (L/S model 7518; Cole-Parmer Instruments Co., Chicago, IL, USA) drives the water around the closed cooling circuit of the Livkit.

The LivKit contains:

- 350 mL of Ablasonic®
- One set of flexible pipes

### Surgical procedure

Anaesthesia will be performed according to standard procedure. Central venous pressure (CVP) will be measured immediately before and after the transection. No pharmacological intervention (such as a diuretic) and no surgical procedure (such as infra-hepatic inferior vena cava clamping) will be used to modify the CVP.

The procedure begins with a full exploration of the abdominal cavity, liver palpation and intra-operative ultrasound to confirm the indication of liver resection. Hepatectomy will be carried out in the same way in the two study arms. In arm A, hepatectomy will be performed after pre-coagulation of the hepatic parenchyma by HIFU along the section plane. This will be the only difference between arm A and arm B. Whenever possible, selective control of inflow will be performed. Otherwise, the Pringle manoeuvre will be used if haemorrhage of more than 500 mL occurs during hepatectomy.

In arm A, pre-coagulation is intended to establish a defined full-thickness plane of coagulative necrosis. This is generally achieved by six to ten side-by-side 40-s HIFU ablations, though more can be used if necessary. Resection is performed in the middle of the pre-coagulated zone.

In both arms, parenchymal division will begin along line marked by electrocautery and then cut with scissors. Liver resection technique (the same for both groups) uses a Kelly clamp claw, a suction device, a bipolar forceps and metallic clips. Blunt clamp dissection is a rapid, easy and cheap method. In essence, a large Kelly clamp is used to crush the liver parenchyma. The relatively soft liver substance dissects away, leaving behind the vascular and biliary structures, which are then ligated by vascular clips.

In arm A, it is planned that all vessels < 5 mm in diameter are sealed successfully using HIFU pre-coagulation and will not require any additional haemostasis. Surgical clips will be used only to control the few vessels > 5 mm in diameter.

After completion of liver parenchyma section and removal of the resected liver, the surgeon will complete haemostasis of the raw surface of the remaining liver. This haemostatic phase will be performed only with Prolene® stitches (Ethicon Inc., Cornelia, GA, USA), or coagulation with electrocautery, bipolar forceps or Argon beam coagulator.

Any use of special haemostatic products [such as Tachosil® (Takeda Pharmaceutical Company Limited, Osaka, Japan) or biological glue] will be noted.

Blood loss volume during this phase of haemostasis will be measured and added to the previous blood loss (during the transection phase) for evaluation of the primary endpoint.

### Data collection and follow-up

**Fig. 2:**
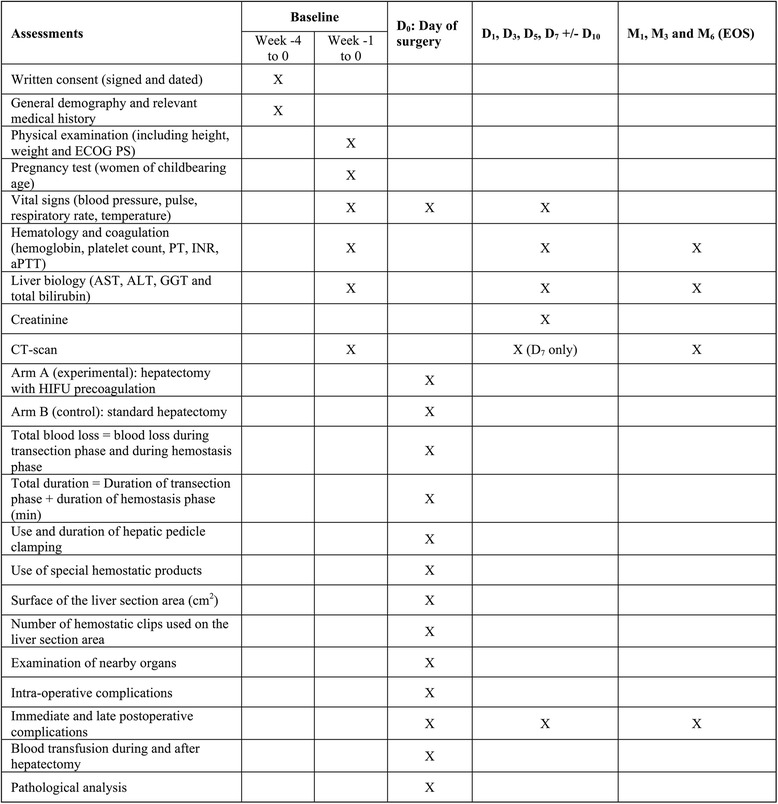
SPIRIT flowchart

Screening/baseline assessments include general demography and relevant medical history, including prior/concomitant therapies and tumour assessment within the 4 weeks before randomization; complete physical examination and laboratory tests within 7 days before randomization. The SPIRIT flowchart of the trial can be found in Fig. 2. Outcomes will be blindly assessed, except for intra-operative measures.

All patients included will be followed up in accordance with the guidelines for patients operated on for CRC LM for up to 6 months post-surgery.

### Statistical considerations

#### Sample size

The study is powered to detect a clinically meaningful reduction in blood loss during hepatectomy in patients with LM. Assuming a blood loss of 7.6 +/− 3.7 mL/cm^2^ in arm B (data based on our own experience), the study will have 85% power to detect a twofold decrease of blood loss in arm A using a Wilcoxon (Mann-Whitney) rank-sum test with a 0.05 two-sided significance level.

Twenty-one randomized patients per arm are required, corresponding to a sample size of 42 patients. Due to the occurrence of potential contraindications to hepatectomy between enrolment and randomization at the beginning of surgery, up to 8 patients may be enrolled in addition to the 42 planned. Indeed, the expected overall number of recruited patients is 50, with an enrolment period of 24 months and an additional follow-up of 6 months after randomization of the last patient.

#### Analysis populations

The three following populations will be defined for statistical analysis:

- The intent-to-treat (ITT) population includes all randomized patients.
- The per protocol (PP) population will consist a subset of patients in the ITT population and includes all patients with no major protocol violation for inclusion and non-inclusion criteria and who are compliant with requirements of the study protocol.
- The safety population includes all patients who underwent the surgery.

All protocol deviations leading to exclusion from the ITT to the PP population will be detailed in the analysis plan.

#### Statistical analyses

Qualitative variables will be described using frequency and percentage distributions. The number of missing data will be given, but will not be considered for the calculation of proportions.

Quantitative data will be described using the number of observations, mean, standard deviation, median, minimum and maximum values.

Patient characteristics and other baseline data (demographics, disease characteristics, clinical and biological data) will be summarized. The date of randomization will serve as a reference for calculation of durations unless otherwise indicated.

All analyses will be performed using the SAS software v9.1 (SAS Institute Inc., Cary, NC, USA).

The primary endpoint will be analysed for the ITT and PP populations.

A Wilcoxon (Mann-Whitney) rank-sum test will be used to compare the normalized blood loss distribution between the two arms at a 5% level in a two-sided situation.

For secondary endpoints, data will be compared between the two arms using a Wilcoxon (Mann-Whitney) rank-sum test. The rate of positive margin and the rate of local recurrence will be summarized by a proportion together with their 95% confidence interval. Post-operative complications will be described by study arm and compared using a Pearson’s chi^2^ test or a Fisher’s exact test, if adequate.

### Data entry and data management

The sponsor (Centre Léon Bérard) will perform the study monitoring and will help the investigators to conduct the study in compliance with the clinical trial protocol, Good Clinical Practice (GCP) and local law requirements.

Data will be entered by investigators on the case report form (CRF). All data will be computerised to detect missing, out-of-range and inconsistent data. Enquiries will be made in the case of erroneous and missing data. Query forms will be sent to the investigators and will be completed in compliance with source data, initialled, dated and sent back to the coordinating centre (Department of Clinical Research of our institution) in charge of data management.

A patient file will be validated once no more inconsistency is detected by the program.

All database modifications will be recorded in the audit trail. The database will be submitted to quality control.

Adverse events will be coded according to the Medical Dictionary for Regulatory Activities (MedDRA®, MSSO, McLean, VA, USA). The database will be locked after all queries are solved, and after data review and final validation.

Computerised management of collected data will be performed by the Department of Clinical Research, using specific tools developed by the Clininfo SA society (Lyon, France).

The sponsor is the owner of all collected data and reports.

### Trial Steering Committee

A steering committee will be composed of sponsor representatives (project manager and statistician manager), the principal investigator and other external participants, as needed. Meetings are scheduled every 3 months from the beginning of the trial. On an ongoing basis, the Steering Committee will review each event that could modify the benefit risk ratio of the study (i.e. scientific, safety, ethics events), all grade ≥ 3 adverse events and adverse events of special interest. Additional meetings may be called at any time if an event occurs or upon request of any members.

The steering committee will be regularly informed of the accrual rate of inclusion and of any emergent problems and will review the activity and safety data throughout the study.

### Independent Data Safety Monitoring Board (iDSMB)

An independent Data Safety Monitoring Board (iDSMB) is convened by the sponsor and composed of experts in the field of liver surgery. Data analyses will be supplied in confidence to the iDSMB, which will be asked to give advice on whether the accumulated data from the trial, together with the results from other relevant research, justifies the continuing recruitment of further patients.

This committee will meet to review each event that could modify the benefit risk ratio of the study (i.e. scientific, safety, ethics events). Meetings are scheduled after the treatment of the first 6 (2 × 3), 12 (2 × 6) and 24 (2 × 12) randomized patients, respectively, to evaluate the safety of the experimental surgical procedure. Additional meetings may be called at any time if an event occurs or on request by one or more members.

The iDSMB will also be regularly informed of the accrual rate of inclusion, each safety issue, and of any emergent problems.

## Discussion

Hepatic resection techniques have been improved using devices such as bipolar forceps, water jet dissectors, ultrasonic cutting systems, and Argon coagulation. These techniques are both expensive and time consuming. Recently, new approaches have been developed to allow bloodless hepatic resection without any vascular exclusion using energy sources such as radiofrequency, ultrasonic scalpels, and microwave coagulation [28–32]. Among these alternative techniques, the use of HIFU has potential advantages.

Preclinical study has demonstrated that HIFU-AR using the new toroidal-shaped transducer during an open procedure in an animal model is safe, reduces bleeding, and can be guided by real-time ultrasound imaging. This work demonstrated blood loss reduction during hepatic resection without the Pringle manœuvre (hepatic pedicle clamping). This was avoided where possible to prevent liver ischemia–reperfusion injury, which is known to predispose patients to post-operative liver failure. In addition, it showed the feasibility, safety, and reproducibility of HIFU-AR during an open procedure. This technique also holds promise in human patients for whom pre-coagulation may offer better control of blood loss, especially in a liver fragile following chemotherapy. Patients undergoing iterative hepatic resection might also benefit from HIFU pre-coagulation. The sample size was calculated to have a twofold decrease in blood loss, because we consider this twofold decrease clinically relevant and in accordance with the results observed in the preclinical study (11.35 mL/cm^2^ versus 4.77 mL/cm^2^). The value chosen for the sample size calculation (7.6 mL/cm^2^ in the control arm) is in the upper limit of reported blood loss in the literature. It is based on personal data when measuring blood loss was determined by aspirated fluid and also by weighing all blood-soaked surgical mops, which is essential, otherwise blood loss might be underestimated.

In the protocol outlined above, the risks are limited since the surgical procedure is identical to the one patients would undergo if not randomized to HIFU-AR. The only difference is that HIFU ablations are carried out on the section plane of the liver. These ablations will be performed only after final checking of the indication for them and immediately before the section phase. The duration of the HIFU ablations will last between 5 and 7 minutes. We consider that this minor increase in operative time will be compensated for by a major decrease in the time necessary to perform liver transection and haemostasis. A potential risk for HIFU-AR patients would be an injury to neighbouring organs secondary to HIFU ablations. Measures taken to avoid this include surgical mops placed under the targeted area to limit risks of ultrasound propagation. Furthermore, this risk was fully assessed during the phase I and IIa studies recently completed and no adverse effects were observed. The post-operative follow-up period will last 6 months, which seems to be sufficient to cover late adverse events. The study participants in the HIFU-AR group can expect to benefit from reduced blood loss and a decreased period of ischemia in the future liver remnant.

Based on previous preclinical and clinical experience, the proposed study design, and the safety monitoring plan described in this protocol, the sponsor considers that adequate risk mitigation measures are included in this protocol and that the benefit-risk profile is favourable.

To limit biases, blood losses are normalized by the resected hepatic area (in square centimetres), which allows to perform several types of anatomical hepatectomy. We also use stratification by surgeon to ensure equal allocation of subgroups of participants (i.e. to each surgeon) in each arm. The three surgeons (AD, MR, PP) are trained with the HIFU prototype, as they already use it in an ongoing study [27].

If the results of this study appear clinically relevant, the development of this technique could be an important improvement in treatment of LM by reducing the invasiveness of surgery. The next step of our research programme is to improve the user interface and then to evaluate the HIFU device in a multicentre phase III study. It may also be possible to extend HIFU-AR to other types of liver resection such as segmentectomy or non-anatomic resections and to primary liver tumours. Furthermore, combining HIFU-AR with HIFU ablation of non-resectable metastases may permit an increase in liver surgery with curative intent for patients with non-resectable LM.

## Trial status

The trial started recruitment in May 2016. The trial is expected to be completed by December 2018.

## Additional file

Additional file 1: SPIRIT checklist. (DOC 120 kb)

## Abbreviations

ANSM: Agence Nationale de Sécurité du Médicament et des Produits de Santé
CPP: Comité de Protection des Personnes
CRC: Colorectal cancer
CRF: Case report form
CVP: Central venous pressure
GCP: Good Clinical Practice
HIFU: High-intensity focused ultrasound
HIFU-AR: HIFU-assisted liver resection
ICH: International Conference for Harmonisation
iDSMB: Independent Data Safety Monitoring Board
INCa: Institut National du Cancer
ITT: Intent-to-treat
LM: Liver metastasis
PP: Per protocol
SPIRIT: Standard Protocol Items: Recommendations for Interventional Trials
